# Surgical Treatment of *Oesophagostomum* spp. Nodular Infection in a Chimpanzee at the CIRMF Primatology Center, Gabon

**DOI:** 10.1155/2021/6617416

**Published:** 2021-03-26

**Authors:** Barthélémy Ngoubangoye, Larson Boundenga, Serge-Ely Dibakou, Thierry-Audrey Tsoumbou, Cyr Moussadji Kinga, Franck Prugnolle, David Fouchet, Dominique Pontier

**Affiliations:** ^1^Centre International de Recherches Médicales de Franceville (CIRMF), BP 769 Franceville, Gabon; ^2^Université de Lyon, Université Lyon 1, CNRS, Laboratoire de Biométrie et Biologie Évolutive UMR 5558, 69622 Villeurbanne, France; ^3^LabEx ECOFECT, Eco-evolutionary Dynamics of Infectious Diseases, University of Lyon, France; ^4^Laboratoire MIVEGEC; UMR-CNRS 5290-IRD 224, IRD Montpellier, France

## Abstract

Oesophagostomosis is a zoonotic disease caused by nematodes of the genus *Oesophagostomum* in the intestinal walls of many species, including ruminants, pigs, humans, and nonhuman primates. Although great apes appear to tolerate the parasite in the wild, they can develop a clinical form that can lead to death in captivity and the natural environment. At the Primatology Centre of the International Centre for Medical Research in Franceville (CIRMF) in Gabon, we recorded 4 deaths of chimpanzees (*Pan t. troglodytes*) caused by *Oesophagostomum* spp. between 2015 and 2019. In each case, coprological analysis was positive for strongylid eggs and abdominal ultrasound revealed nodules about 4 cm in diameter on the intestinal and abdominal walls. Albendazole treatments administered by mouth in two doses of 400 mg six months apart resulted in the disappearance of the parasite in coprological samples but the chimpanzees still died. Autopsies carried out on all four chimpanzees revealed a rupture of the cysts and a discharge of pus into the abdomen in each case. We report surgical management involving the removal of *Oesophagostomum* spp. cysts from a chimpanzee following coprological analysis and abdominal ultrasound examination. Surgical exploration confirmed the fragility of the cystic walls, the rupture of which we avoided. This 5th new case of *Oesophagostomum* ssp. nodules recovered without complications following the operation and could rejoin his group. We suggest that surgical intervention should be considered in similar cases in captive primates, especially chimpanzees.

## 1. Introduction

Oesophagostomosis is a disease caused by parasitic nematodes belonging to the *Oesophagostomum* genus, transmitted by ingestion of L3 larvae found in soiled food or earth. In the infected host, the ingested L3 larvae develop into adult worms whose fertilized females lay eggs that are eliminated in the feces. Sometimes the L3 larvae form nodules in the intestinal wall. The nodule protects the parasite from medical treatment and is the cause of clinical symptoms [1]. This pathology is a human and veterinary public health problem in sub-Saharan Africa where thousands of infections are recorded every year [1–3]. Several *Oesophagostomum* species (*Oesophagostomum bifrustum*, *O. stephanostomum*, and *O. aculeatum*) are common to primates, including humans [4–6]. Infections with *Oesophagostomum* spp. in humans are characterized by symptoms of weight loss, abdominal pain, and diarrhea [7–9] and specific lesions with nodular pathology in the form of several small pea-sized nodules or uninodular masses, called Dapaong tumors [8, 10]. Many studies report the presence of these parasites in chimpanzees, and captive chimpanzees seem to be more infected than their wild conspecifics [1]. With the exception of a few reports of severe clinical symptoms [9, 11, 12], infections are generally asymptomatic [7, 13]. This observation may be due to the little information available for infection in natural environments or because chimpanzees self-medicate by consuming rough leaves which prevents high infestation and consequently severe symptoms [7, 14].

Of various medical protocols, albendazole appears to be the treatment of choice for oesophagostomosis [8, 10, 15]. However, in the case of Dapaong tumors in humans, surgical treatment is often required [8]. At the CIRMF Primatology Centre in Gabon, infection by *Oesophagostomum* spp. caused 4 deaths of chimpanzees (*Pan t. troglodytes*) between 2015 and 2019 despite the systematic use of recommended medical treatments and protocols. Here, we report surgical management involving the removal of *Oesophagostomum* spp. cysts from a chimpanzee following coprological analysis and abdominal ultrasound examination.

## 2. Case Presentation and Management

**Figure 1:**
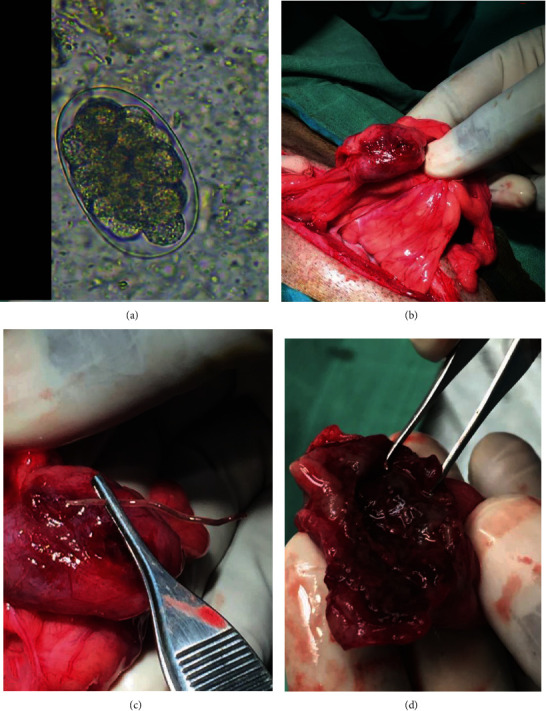
Microscopic image of representative strongylid egg found in feces of Cabinda chimpanzee, of nodules and a worm recovered. (a) Strongylid eggs recovered with the SF-Mc method. (b) Nodules attached on the mesentery. (c) Nodule attached to the descending colon with an adult worm extracted from the nodule. (d) Nodule recovered on the abdominal wall.

Cabinda, a 19-year-old male chimpanzee, was captive-born and weighed 51.3 kg before his illness. He lived at the Primatology Centre of the International Centre for Medical Research in Franceville (CIRMF) in southeast Gabon in a multimale and multifemale group of 21 individuals. In September 2019, Cabinda started to lose weight with episodes of diarrhea and loss of appetite. He was isolated to collect feces. Coprological analyses using sedimentation and flotation methods [16] revealed the presence of strongylid eggs (250 eggs/g of feces) and ultrasound revealed the presence of nodules the size of a small orange (Figure 1) located on mesenteric tissue, the colon and abdominal wall. Albendazole (valbazen®) treatment was administered by mouth in two doses of 400 mg six months apart as recommended in the literature [3], although the use of valbazen® in nonhuman primates was not indicated on the label. A month later, the diarrhea had stopped and no strongyle eggs were detected in fecal analysis. However, Cabinda's clinical condition did not improve, showing lethargy and inappetence. An ultrasound scan showed the same nodules. Given the previous experience of deaths caused by *Oesophagostomum* spp. nodular abscessation, the chimpanzee was anaesthetised, blood samples were taken from the femoral vein to test various hematological and biochemical parameters, and surgery was performed.

Anesthesia was performed (via dart; IM) using a combination of medetomidine hydrochloride 1 mg at 27.4 *μ*g/kg (Domitor®; Orion corporation, Finland) and ketamine hydrochloride 1 g at 1.0 mg/kg (Ketamine® 1 g/ml, Virbac, France). A dose of 1.1 ml of Domitor® and 4 ml of ketamine 1 g ® was administered. Anesthesia was supplemented with Domitor® (0.14 *μ*g/kg) and ketamine 1 g (0.5 mg/kg; IV) to maintain an optimal anesthetic plane. Clinical examination consisted of measuring parameters including general condition, weight (43.1 kg), temperature (37.1°C), oscillation, palpation, and heart rate (94 bpm). Abdominal ultrasound scan revealed 3 large nodules of 4.2 cm x 2.1 cm on mesenteric tissue (Figure 1(b)), 4.1 cm x 2.2 cm on the descending colon (Figure 1(c)), and 3.7 cm x 2 cm on the abdominal wall (Figure 1(d)). Some nodule sizes of peas were located in the intestinal wall. The hematological analyses showed low red blood cells, white blood cells, hematocrit, and elevated monocytes. The biochemistry was within normal limits (Table 1).

After the preparation phase, Cabinda was rehydrated by infusion with alternating solutions of Ringers lactate® and Glucose® 5% (250 ml/h over 2 hours) administered using an 18G catheter. The surgical procedure consisted of removing the nodules after a midline abdominal incision from the pubis to the lower edge of the sternum. Also known as the laparotomy incision, this method allows access to the abdominal organs. The three main nodules were removed due to their large size. The first nodule, located on the mesenteric tissue, was removed by dissection of the tissue and blood vessels ligated with resorbable polyglactin 910 no. 2 thread (5 Ph. Eur, VicrylTM®). The second nodule in the wall of the descending colon was removed by incision around the cyst. The wall of the colon was sutured with resorbable polyglactin 910 no. 2 suture (5 Ph. Eur, VicrylTM®) to avoid adhesions during healing. It was checked that the contents of the colon did not fall into the abdomen. The third nodule was removed from the abdominal wall by dissection.

Nodule walls were necrotic and fragile (Figure 1(d)) and the nodules on the mesenteric tissue (Figure 1(b)) and the descending colon, ruptured during the operation. The nodule on the abdominal wall was removed entirely (Figure 1(d)). Each of the 3 large nodules contained a worm but all were dead (Figure 1(c)). The abdomen was cleaned with Ringer Lactate fluid and sutured using polyglactin 910 suture no. 2 (5 Ph. Eur, VicrylTM®). The overlock method was used to suture the abdominal muscles and single stitches to suture the skin.

Cabinda's postoperative management included antibiotics (amoxicillin, Duphamox LA®) at 15 mg/kg body mass and an analgesic (buprenorphine, Vetergesic®, Inject Care Parenterals) at 15 *μ*g/kg body mass. Relay therapy was administered 48 hours later with amoxicillin/clavulinic acid (Augmentin®) 40 mg/5 mg/kg daily in three doses for one week and ibuprofen 400 mg (Advil®) in two doses for three days. Cabinda recovered well and no complications were noted. After two days of liquid food, he resumed normal eating and presented a good general condition. At a check-up, two months later, he weighed 53.20 kg.

## 3. Discussion

Oesophagostomosis is a disease described in several primate species including humans [1, 17]. In chimpanzees, both in the wild and in captivity, the disease is marked by various degrees of pathogenicity including weight loss, appetite loss, diarrhea, and death in some cases [1, 7, 12, 18]. Some authors mention the ingestion of rough leaves in wild primates to expel parasites and prevent serious infestations [1, 7], and stress linked to captivity could also partly explain the differences in tolerance of *Oesphagostomum* spp. infections between in wild and captive animals.

Albendazole is the treatment of choice in both humans and other primates for medical treatment of *Oesophagostumum* infections [8, 10]. Therapeutic regimens are varied but many studies recommend the protocol used in this case: 400 mg in two doses six months apart [1, 3]. However, since 2015, despite the systematic use of this protocol on 68 infected primates (*Mandrillus sphinx* (*N* = 26), *Cercopithecus cephus* (*N* = 4), *Cercopithecus nictitans* (*N* = 6), *Macaca* sp. (*N* = 13), and chimpanzees (*N* = 19)), we have recorded 4 cases of death in 6 chimpanzees with large *Oesophagostumum* spp. nodules detected using ultrasound when coprological analyses carried out after treatment were negative for strongylid eggs. In contrast, 13 chimpanzees presenting pea-sized cysts regained their health following treatment. No other infected species developed large cysts. These observations confirm (i) the sensitivity of chimpanzees to *Oesophagostumum* spp. infection and show that albendazole is effective on nonencysted parasites and small cysts but ineffective on parasites protected in large nodules. Medical treatment alone would therefore not be sufficient when the nodules are large. (ii) At an advanced stage of development, it is possible for the parasite to die in the nodule but the walls of the nodules are so fragile that they rupture even after the death of the parasite. This hypothesis is supported by the observations that the adult worms we found in Cabinda's cysts were dead (Figure 1(c)), but the nodule walls were necrotic and so fragile (Figure 1(d)) that they ruptured during the operation (Figure 1(d)). In addition, during autopsies, we found ruptured nodules in three dead chimpanzees between 2015 and 2019. As pus is produced by pyogenic organisms [19], the leakage of pus into the abdomen may lead to the release of toxins or cause septicemia or anaphylactic shock leading to death [20]. (iii) The size of the nodules may constitute an obstacle to the intestinal transit which can also lead to death [1].

Thus, in the case of Cabinda, although the coprological analyses were negative after treatment and hematology showed only mild changes, there was a risk of nodule abscessation and rupture; therefore, surgical treatment was elected. The combination of medical and surgical treatment was successful in achieving full clinical resolution. Treatment by surgery was motivated by the fact that, in humans, the treatment of oesophagostomosis is also surgical in cases of Dapaong tumors [8]. Thus, unlike the four other chimpanzees who died, the results obtained for Cabinda are encouraging. He regained his appetite, good general condition, and his body mass increased from 49.9 kg to 53.2 kg in only two months after the operation. Today, he has regained its group and is leading a normal life. No complications have been reported.

*Oesophagostumum* spp. infections are common at the CIRMF primatology center and 5 cases of nodular pathology, including 4 deaths, have been registered since 2015. A preventive schedule consisting of systematic treatment with albendazole 400 mg (Valbazen®) every 6 months [3] has been implemented. In addition, coprological analyses for strongylid eggs will be carried out automatically every three months. Positive subjects for strongylid eggs will receive a new dose of Albendazole (Valbazen®) and further coprological analyses will be performed. The combination of these measures should reduce the high prevalence of *Oesophastomum* spp. in the centre and above all prevent nodular pathology.

## 4. Conclusion and Recommendations

This experience leads us to recommend that for captive primates and chimpanzees, in particular, surgical extraction of *Oesophagostumum* nodules should be performed in addition to medical treatment when the nodules are large. Furthermore, because of the zoonotic nature of the infection and the close phylogenetic relationship between humans and great apes that result in opportunities for parasite exchange [21], an ultrasound examination is systematically recommended during health checkups of personnel working with primates or living in risk areas, when coprological analyses are positive for strongylid eggs.

## Figures and Tables

**Table 1:** Hematologic and serum biochemical analyses for chimpanzee Cabinda. Reference values are those applied at the CIRMF Primatology Center (unpublished data on 650 samples from 156 adult male chimpanzees over 15 years).

Type of analyses	Analytes	Cabinda values	Reference values
Hematology	Red blood cells	4.2 x 10^6^/mm^3^	5.23 ± 0.62 x 10^6^/mm^3^
	White blood cells	4.6 x 10^3^/mm^3^	9.8 ± 3.8 x 10^3^/mm^3^
	Platelet	340 x 10^3^/mm^3^	259 ± 80 x 10^3^/mm^3^
	Monocytes	11.3 %	1.49 +/-2.03 (%)
	Lymphocytes	56.3 %	46+/-17 (%)
	Hemoglobin	12.4 (g/dl)	14+/-1.6 g/dl
	Hematocrit	35.8 (%)	43+/-2 (%)

Serum biochemistry	Triglycerides (md/dl)	0.46 g/l	0.49 +/- 0.15 (g/l)
	Total protein (g/dl)	88 g/l	76 +/- 4 (g/l)
	Creatinine (mg/dl)	110 *μ*mol/l	91.6 +/-21.7 *μ*mol/l
	Blood urea nitrogen (mg/dl)	5.8 mmol/l	2.5 +/- 2.0 mmol/l
	Alanine transaminase (ALT; IU/l)	31 (IU/l)	26+/- 10 IU/l
	Aspartate transaminase (AST)	42 (IU/l)	34+/-17 IU/l
	Alkaline phosphatase	69 (IU/l)	321+/- 288 IU/l
